# Supplementary material for: Altered muscle niche contributes to myogenic deficit in the D2-mdx model of severe DMD
Source: Cell Death Discov. 2023 Jul 4;9:224. doi: 10.1038/s41420-023-01503-0 (PMC10319851; doi:10.1038/s41420-023-01503-0)
Supplement: Supplementary file 1 — Supplemental Material [file 41420_2023_1503_MOESM1_ESM.pdf]

Supplemental Table 1. Primary and secondary antibodies for immunostaining.

Protein Target	Primary Antibody	Secondary Antibody
BrdU	Anti–BrdU-biotin, Life Technologies, B35138 (1:100)	Streptavidin Alexa Fluor 488, (1:500), Thermo Fisher, S32354<br>Streptavidin Alexa Fluor 568, (1:500), Thermo Fisher, S11226
eMHC	Anti-eMHC, DSHB, F1.652 (1:25)	Goat anti-mouse IgG1 Alexa Fluor 488, (1:500), Thermo Fisher, A-21121
F4/80	Anti-F4/80; Bio-Rad, MCA497R (1:100)	Goat anti-rat IgG (H+L) Alexa Fluor 488, (1:500), Thermo Fisher, A-11006<br>Goat anti-rat IgG (H+L) Alexa Fluor 647, (1:500), Thermo Fisher, A-21247
iNOS	Anti-iNOS, Thermo Fisher, PA3-030A (1:100)	Goat anti-rabbit IgG (H+L) Alexa Fluor 488 (1:500), Thermo Fisher, A-11008<br>Goat anti-rabbit IgG (H+L) Alexa Fluor 568 (1:500), Thermo Fisher, A-11011
CD206	Anti-CD206, Bio-Rad, MCA2235 (1:50)	Mouse Anti-rat IgG2a Alexa Fluor 488 (1:250), Abcam, ab172332
PDGFRα	Anti-PDGFRα, Cell Signaling, D1E1E (1:100)	Goat anti-rabbit IgG (H+L) Alexa Fluor 488 (1:500), Thermo Fisher, A-11008<br>Goat anti-rabbit IgG (H+L) Alexa Fluor 568 (1:500), Thermo Fisher, A-11011
Laminin-α2	Anti-laminin-α2 (4H8-2); Sigma, L0663 (1:250)	Goat anti-rat IgG (H+L) Alexa Fluor 488 (1:500), Thermo Fisher, A-11006<br>Goat anti-rat IgG (H+L) Alexa Fluor 647 (1:500), Thermo Fisher, A-21247

Description of protein target, primary antibody (manufacturer, catalog number, dilution) and secondary antibody (Alexa Fluor conjugation, manufacturer, catalog number, dilution) shown for all immunostaining procedures performed in this study.

Supplemental Table 2. Taqman assays for quantitative reverse transcriptase PCR (qRT-PCR).

Gene Target	Gene Name	Taqman Assay
Pax7	Paired box 7	Mm01354484_m1 [FAM-MGB], Thermo Fisher
Myog	Myogenin	Mm00446194_m1 [FAM-MGB], Thermo Fisher
Myod1	Myogenic differentiation 1	Mm00440387_m1 [FAM-MGB], Thermo Fisher
Spp1	Secreted phosphoprotein 1	Mm00436767_m1 [FAM-MGB], Thermo Fisher
Tnf	Tumor necrosis factor	Mm00443258_m1 [FAM-MGB], Thermo Fisher
Arg1	Arginase	Mm00475988_m1 [FAM-MGB], Thermo Fisher
Postn	Periostin	Mm01284919_m1 [FAM-MGB], Thermo Fisher
Il10	Interleukin 10	Mm01288386_m1 [FAM-MGB], Thermo Fisher
Nos2	Nitric oxide synthase 2	Mm00440502_m1 [FAM-MGB], Thermo Fisher
Il1b	Interleukin 1 beta	Mm00434228_m1 [FAM-MGB], Thermo Fisher
Il6	Interleukin 6	Mm00446190_m1 [FAM-MGB], Thermo Fisher
Cd163	CD163 antigen	Mm00474091_m1 [FAM-MGB], Thermo Fisher
Fn1	Fibronectin 1	Mm01256744_m1 [FAM-MGB], Thermo Fisher
Col1a1	Collagen type I alpha 1	Mm00801666_g1 [FAM-MGB], Thermo Fisher

Gene symbol, gene name and corresponding Taqman assay for all qRT-PCR procedures performed in this study.

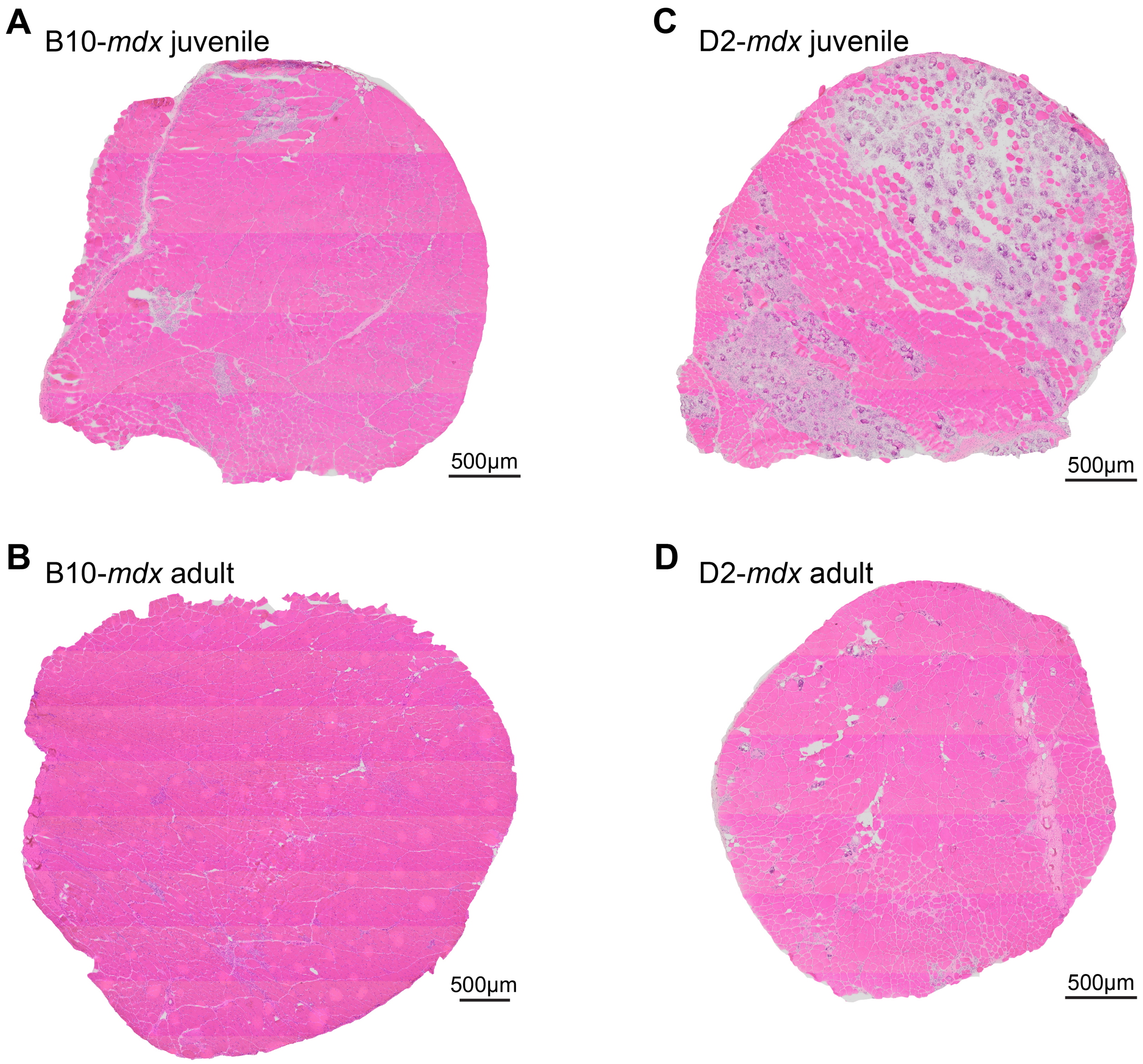

Supplemental Fig. 1. Histopathology in D2-*mdx* and B10-*mdx* models with age. A-D. Whole cross-sectional H&E staining of triceps harvested from juvenile and adult B10-*mdx* (**A**, **B**, respectively) and D2-*mdx* B10-*mdx* (**C**, **D**, respectively) mice.
